# Immune Infiltration Represents Potential Diagnostic and Prognostic Biomarkers for Esophageal Squamous Cell Carcinoma

**DOI:** 10.1155/2022/9009269

**Published:** 2022-06-26

**Authors:** Mengxiang Li, Pan Chen, Yuanji Zhao, Xiaoshan Feng, Shegan Gao, Yijun Qi

**Affiliations:** ^1^School of Information Engineering of Henan University of Science and Technology, 263 Kaiyuan Road, Luoyang 471023, China; ^2^Henan Key Laboratory of Microbiome and Esophageal Cancer Prevention and Treatment, Henan Key Laboratory of Cancer Epigenetics, Cancer Hospital, The First Affiliated Hospital, College of Clinical Medicine of Henan University of Science and Technology, Luoyang 471003, China; ^3^College of Biological Sciences, University of California Davis, 1 Shields Ave, Davis, CA 95616, USA

## Abstract

**Background:**

Immune infiltrates in the tumor microenvironment have established roles in tumor growth, invasion, and metastasis. However, the diagnostic and prognostic potential of immune cell signature in esophageal squamous cell carcinoma (ESCC) remains unclear.

**Results:**

The proportions of 22 subsets of immune cells from 331 samples including 205 ESCC and 126 normal esophageal mucosa retrieved from TCGA, GEO, and GTEx databases were deciphered by CIBERSORT. Nine overlapping subsets of immune cells were identified as important features for discrimination of ESCC from normal tissue in the training cohort by LASSO and Boruta algorithms. A diagnostic immune score (DIS) developed by XGBoost showed high specificities and sensitivities in the training cohort, the internal validation cohort, and the external validation cohort (AUC: 0.999, 0.813, and 0.966, respectively). Furthermore, the prognostic immune score (PIS) was developed based on naive B cells and plasma cells using Cox proportional hazards model. The PIS, an independent prognostic predictor, classified patients with ESCC into low- and high-risk subgroups in the internal validation cohort (*P* = 0.038) and the external validation cohort (*P* = 0.022). In addition, a nomogram model comprising age, N stage, TNM stage, and PIS was constructed and performed excellent (HR = 4.17, 95% CI: 2.22-7.69, *P* < 0.0001) in all ESCC patients, with a time-dependent 5-year AUC of 0.745 (95% CI: 0.644 to 0.845), compared with PIS or TNM stage as a prognostic model alone.

**Conclusion:**

Our DIS, PIS, and nomogram models based on infiltrated immune features may aid diagnosis and survival prediction for patients with ESCC.

## 1. Introduction

Esophageal cancer (EC) remains one of the most common malignant tumors worldwide and ranks seventh and sixth among all malignant tumors in morbidity and mortality, respectively [1, 2]. Although esophageal adenocarcinoma (EAC) is dominant in the United States, Europe, and other western countries, esophageal squamous cell carcinoma (ESCC) comprises more than 90% of EC cases in China [3]. Despite recent advances in diagnostics and therapeutics, the 5-year overall survival rate of EC remains 15-25% largely due to the lack of screening measures for early diagnosis and effective therapeutic regimens [4, 5]. The majority of ESCC have metastatic disease at initial diagnosis, leading to futile clinical management [6]. As such, it is of utmost importance to identify biomarkers for early detection, diagnosis, prognosis, and therapeutic intervention of ESCC as well.

Genomic, epigenomic, and proteomic alterations intrinsic to cancer cells have been extensively investigated and identified as driver agents in development and progression of ESCC [7–9]. Notwithstanding the clinical relevance of these molecular features, few dysregulations are clinically targetable in clinical care of ESCC. Instead, accumulating evidence also demonstrates the tumorigenic role of cancer-cell-extrinsic factors [10]. The esophageal tumor tissues are populated with a great variety of stromal and immune cell types that exert both pro- and anti-tumorigenic effects. In ESCC, multiple studies have reported correlations between prognosis and immune cells and stromal components [11]. Furthermore, autoantibodies against a panel of 6 tumor-associated antigens show good performance for discrimination of early-stage ESCC from normal controls, which indicates ESCC-specific immune response arising in the setting of ESCC [5]. The tumor-infiltrating immune cells are correlated with invasion, metastasis, tumor stage, poor prognosis, and therapeutic outcomes.

The commonly used methods including immunohistochemistry, immunofluorescence, flow cytometry, and cytometry by time-of-flight mass spectrometry can only characterize limited types of immune cells based on preselected cellular markers, such as CD8 cells, Treg cells, Th17 cells, and tumor-associated macrophages (TAMs), thus providing limited knowledge of the collective effects of these heterogeneous immune cells [12]. The tumor fate, however, is dictated by numerous specialized cell types that interact in a highly coordinated manner [13–16]. Although information of individual cell types was kept in bulk transcriptomics data from tumor tissues, it is challenging to decipher the individual cellular identity mingled together. To estimate the immune cell proportions from bulk tumor samples, multiple computational methods have been developed [12, 17]. For example, CIBERSORT is a computational algorithm to enumerate the relative proportions of immune cells using transcriptional data from bulk tumor tissues [18].

In the present study, CIBERSORT was used to enumerate the immune cellular composition of ESCC based on bulk transcriptome data of ESCC and normal esophageal mucosa samples from Gene Expression Omnibus (GEO), Genotype-Tissue Expression (GTEx), and The Cancer Genome Atlas (TCGA). Diagnostic and prognostic models were developed and validated with good performance.

## 2. Materials and Methods

### 2.1. Patients and Datasets

This study used data in the public domain. The transcriptome data GSE53625/GSE23400 that comprise 179/53 human ESCC samples together with adjacent normal tissue samples and clinical data were downloaded from GEO (https://www.ncbi.nlm.nih.gov/geo/). The gene expression data of 92 ESCC samples from TCGA and 338 normal esophageal mucosa tissue samples from GTEx were derived from UCSC Xena (https://xena.ucsc.edu/). The expression level of mRNA in TCGA and GTEx were normalized to log_2_(TPM + 0.001) (TPM (transcripts per kilobase of exon model per million mapped reads)) to improve the representation.

For prognostic analysis, eligible subjects were recruited according to the following criteria: (1) histology confirmed diagnosis of ESCC and (2) available follow-up of ≥3 months and prognostic information. As such, 101 ESCC patients from GSE53625 and 51 patients from TCGA were included in the present study. One hundred one patients with ESCC were randomly divided into the training cohort (71 patients) and internal validation cohort (30 patients). Patients with ESCC from TCGA were used as an external validation cohort.

### 2.2. Estimation of Immune Cell Infiltration

To quantify the proportions of immune cells, the current study utilized the CIBERSORT algorithm (http://cibersort.stanford.edu/), which is a deconvolution algorithm to estimate the proportions of 22 immune cell phenotypes based on a gene expression signature matrix of 547 genes representing each of 22 cells. These 22 infiltrating immune cells include naive B cells, memory B cells, plasma cells, CD8 T cells, naive CD4 T cells, resting memory CD4 T cells, activated memory CD4 T cells, follicular helper T cells, regulatory T cells, gamma delta T cells, resting NK cells, activated NK cells, monocytes, M0 macrophages, M1 macrophages, M2 macrophages, resting dendritic cells, activated dendritic cells, resting mast cells, activated mast cells, eosinophils, and neutrophils. CIBERSORT yields a *P* value for each sample using Monte Carlo sampling, providing a measure of confidence in the results. Generally, samples with *P* < 0.05 indicate that the inferred fractions of immune cells calculated by CIBERSORT were considered eligible for further analysis.

ESTIMATE (Estimation of Stromal and Immune Cells in Malignant Tumor Tissues using Expression data) algorithm generates the immune score that represents the infiltration of immune cells in tissue, based on single sample gene set enrichment analysis using gene expression data [19]. Several reports have demonstrated that immune scores and stromal scores produced by ESTIMATE algorithm could separate normal cells from tumor cells through analyzing specific gene expression signature of immune and stromal cells [20–22]. The ESTIMATE outputs were reduced by a factor of 1000 to be comparable with the CIBERSORT outputs.

### 2.3. Feature Selection

The objective of feature selection is to identify the specific factors that are most effective in discriminating normal from cancerous tissues. Reduction of feature number can alleviate the problem of overfitting. Another important advantage of feature selection rather than other dimensionality reduction techniques, such as principal component analysis and wavelet transform, is that the original features are maintained. Eligible samples were randomly separated into the training and validation cohorts (7 : 3) using the “Sample” function in R software. Two feature selection approaches including LASSO and Boruta were used to assess the importance of intratumor infiltrated immune cells [23, 24]. LASSO minimizes the sum of squared errors for ranking and selecting variables in statistical models. Boruta is usually used for feature selection from all relevant features on the basis of random forest (RF) classifier.

### 2.4. Classifier Development

XGBoost (eXtreme gradient boosting) [25, 26] is an ensemble learning algorithm based on gradient boosting tree and provides state-of-the-art results for many bioinformatics problems. It uses the gradient boosting framework and provides a parallel tree boosting technique, which can solve a variety of problems with high accuracy. The super parameters of XGBoost were determined by grid search and 10-fold cross-validation, including the number of iterations (nrounds = 200), step size shrinkage used to prevent overfitting (eta = 0.15), maximum depth of a tree (max depth = 3), minimum loss reduction required to make a further partition on a leaf node of the tree (gamma = 0.25), parameters for ratio of random subsampling characteristic (colsample bytree = 0.2), and minimum sum of instance weight (hessian) needed in a child tree (min child weight = 0.7). Compared with other machine learning algorithms, XGBoost has certain unique advantages. The most important strengths are that XGBoost performs a second-order Taylor expansion for the objective function and uses the second derivative to accelerate the convergence speed of the model while training. Subsequently, we performed survival analysis to obtain robust survival-associated immune cells from that selected by diagnostic classifier.

In this study, the predictive model was implemented by an R package called XGBoost (version 1.3.2.1), available from https://cran.r-project.org. The parameters of XGBoost can be optimized by grid search method with cross-validation in the training cohort.

### 2.5. Performance Evaluation Metrics

To objectively evaluate the performance of classifier, the following metrics, including sensitivity (Sn), specificity (Sp), and overall accuracy (Acc), are used in this study and calculated as follows:

(1) $$\begin{array}{c} \text{Sn}=\frac{\text{TP}}{\text{TP}+\text{FN}},\;0\leq \text{Sn}\leq 1,\\ \text{Sp}=\frac{\text{TN}}{\text{TN}+\text{FP}},\;0\leq \text{Sp}\leq 1,\\ \text{Acc}=\frac{\text{TP}+\text{TN}}{\text{TP}+\text{FP}+\text{TN}+\text{FN}},\;0\leq \text{Acc}\leq 1, \end{array}$$

where TP, TN, FP, and FN indicate the true positives, true negatives, false positives, and false negatives, respectively. The values of Acc, Sn, and Sp reflect the robustness of the classifiers.

In addition, the receiver operating characteristic (ROC) curve plots the signature performance of true positive rate (TPR = sensitivity) against false positive rate (FPR = 1 − specificity). The area under the ROC curve (AUC) is also used as performance evaluation in this study, which can quantitatively and objectively measure the performance of the proposed classifier. A perfect predictor is proved to have an AUC = 1, and the random performance is AUC = 0.5.

### 2.6. Statistical Analysis

For each immune cell fraction, we calculated the 75% quartile, median, and 25% quartile of the normal and tumor groups. Group comparisons for continuous variables were performed using Wilcoxon-signed rank test or Student's *t*-test. Correlation analysis was performed by package “corrplot” of R. The LASSO analysis was carried out using “glmnet” package. Survival ROC was plotted using “survivalROC” package. Decision curve analysis was carried out with “rmda” package. A nomogram and calibration plots were developed with “rms” package. Kaplan-Meier survival analyses with log-rank tests were applied using the “survival” package. Time-dependent ROC (survival ROC) curves were applied to assess the prognostic power of nomogram risk score. The above statistical analyses were conducted using R software 4.1.0. All statistical tests were two-tailed, and *P* < 0.05 was considered statistically significant.

## 3. Results

### 3.1. Eligible Samples

The present study involved 4 source datasets, including GSE53625, GSE23400, TCGA-ESCC, and GTEx. The overall proportions of immune versus nonimmune cells were estimated by CIBERSORT algorithm. As the CIBERSORT *P* values anticorrelate with the abundance of immune cells in bulk tissues, *P* < 0.05 was used as a threshold to select the eligible samples for further analysis.

As such, 101 ESCC and 53 normal samples from GSE53625, 53 ESCC and 48 normal samples from GSE23400, 51 ESCC samples from TCGA, and 25 normal esophageal mucosa samples from GTEx were eligible for subsequent analysis. Demographic and clinicopathological characteristics of patients with ESCC in GSE53625 and TCGA-ESCC (the corresponding information unavailable for subjects from GSE23400 and GTEx) are shown in Table 1.

### 3.2. Proportions of Infiltrated Immune Cells

**Figure 1:**
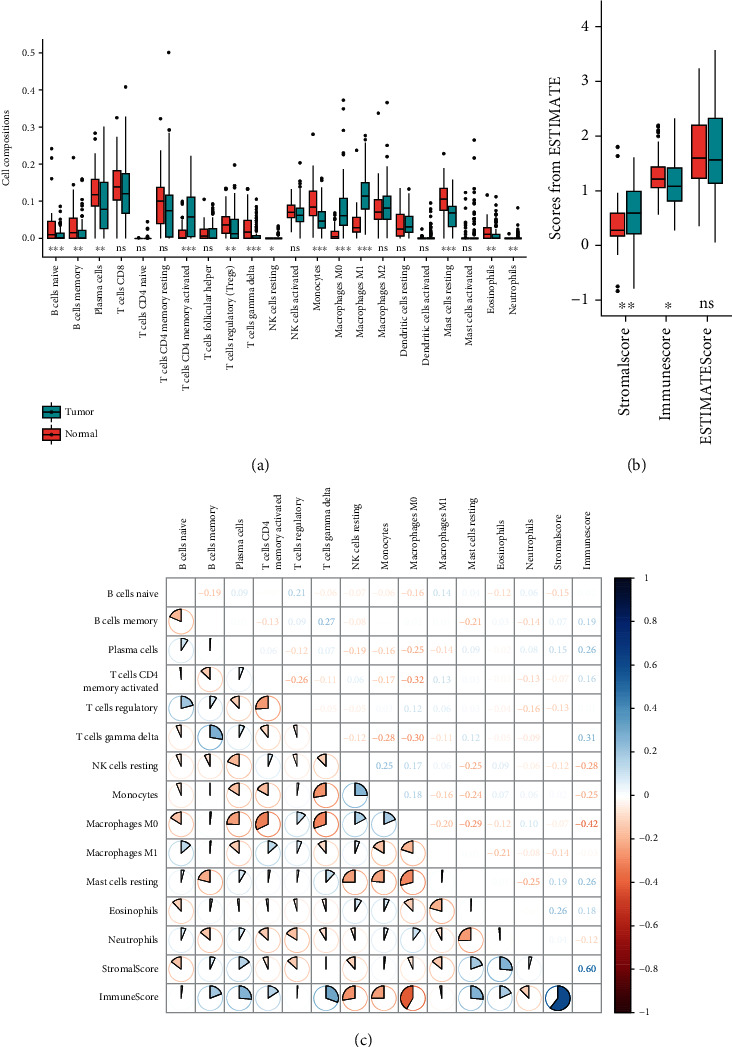
Distribution and correlations of immune cells between tumor and normal tissue. (a) Box plot of 22 immune cell types from CIBERSORT between normal and tumor tissues. The blue and red colors represent tumor and normal tissues, respectively. Inner box plot shows the 75% quartile, median, and 25% quartile. (b) Box plot of 3 scores from ESTIMATE. (c) Correlation analyses among all 13 immune cells as well as StromalScore and ImmuneScore in ESCC samples from GSE53625. The blue and red colors indicate the positive and negative correlations, respectively. *P* values were calculated by the Wilcoxon-signed rank test. ns: *P* ≥ 0.05; ∗: *P* < 0.05; ∗∗: *P* < 0.01; ∗∗∗: *P* < 0.001.

The proportions of 22 immune cells in ESCC and normal tissues evaluated by CIBERSORT algorithm are shown in Supplemental Figure 1. After Wilcoxon-signed rank test analysis, 13 of the 22 immune cells exhibited statistical differences between ESCC and normal tissues. The proportions of activated memory CD4 T cells, M0 macrophages, M1 macrophages, and neutrophils were significantly higher in tumor tissues, whereas naive B cells, memory B cells, plasma cells, regulatory T cells (Tregs), gamma delta T cells, resting NK cells, monocytes, resting mast cells, and eosinophils were less abundant in tumor tissues than in normal tissues (Table 2 and Figure 1(a)).

In addition, ESTIMATE was used to calculate the scores of StromalScore, ImmuneScore, and ESTIMATEScore for each sample. We found that the StromalScore was significantly increased in ESCC samples, whereas the ImmuneScore was significantly decreased in ESCC samples (Table 2 and Figure 1(b)). Furthermore, the correlation between StromalScore and ImmuneScore was significant, with a correlation coefficient of 0.60.

Correlations among all 13 immune cells as well as StromalScore and ImmuneScore are shown in Figure 1(c). We observed positive correlations between gamma delta T cells and ImmuneScore and StromalScore and ImmuneScore with the correlation coefficients greater than 0.3. The negative correlation categories comprised M0 macrophages and activated memory CD4 T cells, M0 macrophages and gamma delta T cells, and M0 macrophages and ImmuneScore, with coefficient less than -0.3.

In addition, increased proportions of plasma cells and resting mast cells, decreased proportion of follicular helper T cells, and increased scores of StromalScore and ESTIMATEScore were found in male patients. In the category of alcohol use, the proportions of memory B cells, plasma cells, and gamma delta T cells were significantly decreased in ESCC patients with alcohol exposure (Supplemental Figure 2). With regard to T stage category, the higher the T stage was, the higher scores of StromalScore, ImmuneScore, and ESTIMATEScore were found in ESCC. In categories of N stage and TNM stage, the percentage of gamma delta T cells was positively correlated with disease status (Supplemental Figure 3).

### 3.3. Candidate Features as Biomarker for ESCC

**Figure 2:**
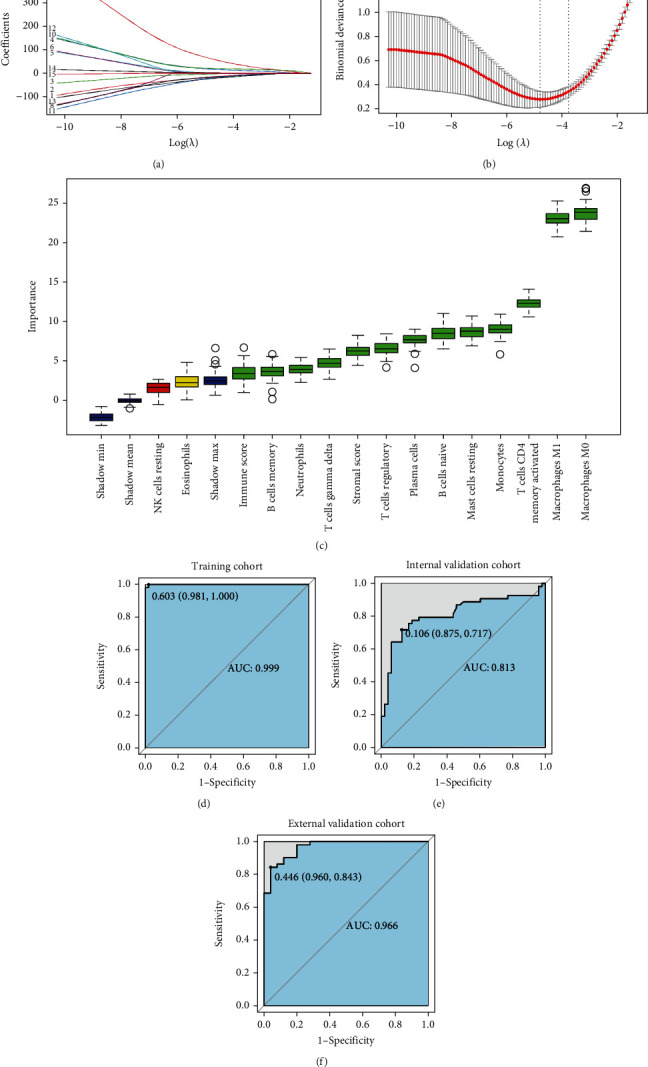
Feature selection and predictive performance of the DIS. (a) LASSO coefficient profiles of the fractions of 15 immune cell types. (b) Tenfold cross-validation for parameter selection in the LASSO model. (c) Results of Boruta analysis in the training cohort. Color coding according to green = selected, yellow = tentative, and red = rejected. ROC curves of DIS in the (d) training, (e) internal validation, and (f) external validation cohorts. Note: DIS: diagnostic immune score; AUC: area under the ROC curve.

Using 101 ESCC and 53 normal samples from GSE53625 as the training cohort, the proportions of 13 immune cells and 2 ESTIMATEScores, derived from CIBERSORT and ESTIMATE, respectively, were used as input features for classification of ESCC and normal tissues. These features were subjected to importance evaluation by two feature selection algorithms, LASSO and Boruta. The 15 selected features in order of importance by LASSO comprised naive B cells, memory B cells, plasma cells, activated memory CD4 T cells, monocytes, M0 macrophages, M1 macrophages, resting mast cells, and StromalScore (Figures 2(a) and 2(b)).  Figure 2(c) shows that 13 green features identified by Boruta algorithm contributed significantly to classification of ESCC. These 13 candidate features comprised naive B cells, memory B cells, plasma cells, activated memory CD4 T cells, regulatory T cells (Tregs), gamma delta T cells, monocytes, M0 macrophages, M1 macrophages, resting mast cells, neutrophils, StromalScore, and ImmuneScore. The 9 common features identified by both LASSO and Boruta algorithm were deemed as candidate biomarkers for ESCC in this study, which were exactly equivalent to features selected by LASSO. The correlations of these 9 features are shown in Figure 1(c), in which M0 macrophages were strongly correlated with activated memory CD4 T cells with a correlation coefficient of -0.32. The correlations between other features were not significant.

### 3.4. Diagnostic Signature for ESCC

In the training cohort of 101 ESCC tissues and 53 normal tissues, we calculated the diagnostic immune score (DIS) using XGBoost method. For differentiation of ESCC from normal tissues, the cutoff score of DIS was determined by ROC curve. Using a DIS cutoff score of 0.603, an AUC of 0.999 was attained for classification of 154 tissue samples in the training cohort, with Sn and Sp of 0.981 and 0.999, respectively (Figure 2(d), Table 3). In the internal validation cohort of 53 ESCC tissues and 48 normal tissues from GSE23400, the DIS also showed robust performance for discrimination of ESCC from tissue samples with an AUC of 0.813 (Figure 2(e)). Consistently, in the external validation cohort (51 ESCC tissue from TCGA-ESCC and 25 samples from GTEx), the AUC was 0.966 (Figure 2(f)). Our data highlights that both immune infiltrate and nonimmune stromal components have clinical implication for ESCC diagnosis.

### 3.5. Prognostic Signature for ESCC

**Figure 3:**
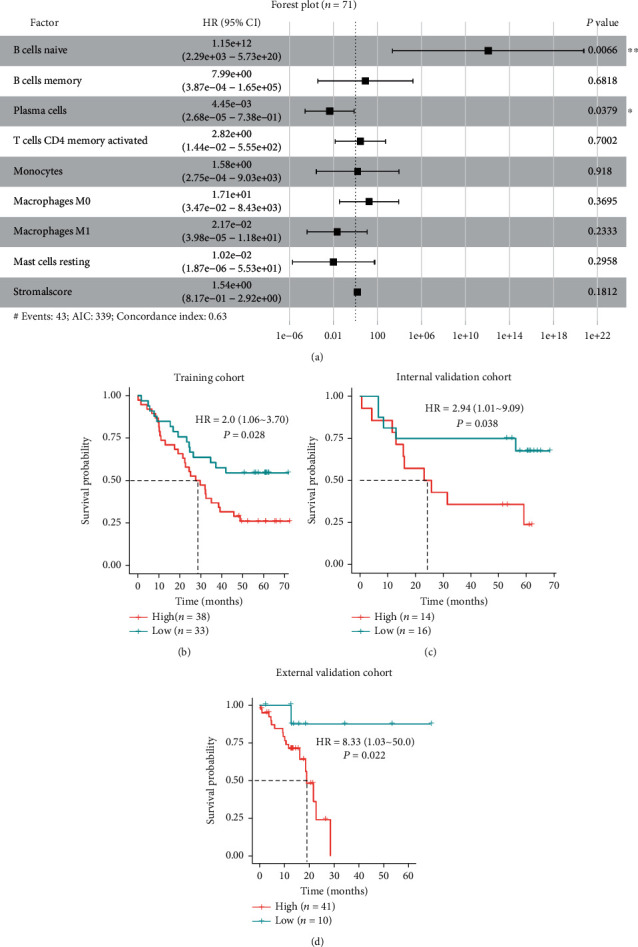
Multivariate Cox proportional hazard regression analysis and survival analysis. (a) Forest plots show significant survival-related immune cells based on multivariate Cox regression analyses. Kaplan-Meier curves for overall survival grouped by PIS in the (b) training, (c) internal validation, and (d) external validation cohorts. Note: *P* values were calculated by log-rank test. HR: hazard ratio; CI: confidence interval; PIS: prognostic immune score. ∗: *P* < 0.05; ∗∗: *P* < 0.01.

All 101 ESCC patients with survival data were randomly assigned to the training (70%, with 71 samples) and validation (30%, with 30 samples) cohorts in the present study. In the training cohort, naive B cells, M0 macrophages, resting mast cells, and StromalScore were significant prognostic factors among the 9 candidate biomarkers for ESCC by univariate Cox proportional hazard regression analysis (Supplemental Table 1). Multivariate Cox proportional hazard regression analysis showed that naive B cells and plasma cells were independent prognostic factors for patients with ESCC after adjusting potential confounding factors (Figure 3(a)). For risk score calculation by integrating the independent prognostic factors, the prognostic immune score (PIS) for each individual was calculated using Cox proportional hazards model in the training cohort. The formula for PIS calculation was as follows: PIS = (naive B cells × 27.71) + (plasma cells × (−5.50)).

The cutoff value of PIS for prognostic predication of patients with ESCC was determined using the “survminer” package. Using a cutoff value of PIS of -0.357, ESCC patients in the training cohort were divided into the high- and low-PIS groups. The Kaplan-Meier survival analysis showed that the median survival times of high-PIS and low-PIS subgroups were 28.6 months and >60 months, respectively (Figure 3(b)). Log-rank test showed that the survival times of ESCC patients in these two groups were significantly different, with a hazard ratio of 2.0 for patients with high PIS (95% CI: 1.06 to 3.70, *P* = 0.028, Figure 3(b)), and similar results were observed in the internal validation cohort (HR = 2.94, 95% CI: 1.01 to 9.09, *P* = 0.038, Figure 3(c)) and in the external validation cohort (HR = 8.33, 95% CI: 1.03 to 50.0, *P* = 0.022, Figure 3(d)).

The Kaplan-Meier survival analysis of esophagus adenocarcinoma (EAC) cohort in TCGA showed that there was no significant difference between the low- and high-PIS groups (HR = 2.04, 95% CI: 0.564 to 7.69, *P* = 0.26, Supplemental Figure 4(a)), consistent with the distinctive molecular phenotypes manifested by ESCC and EAC.

### 3.6. Prognostic Nomogram for ESCC

**Figure 4:**
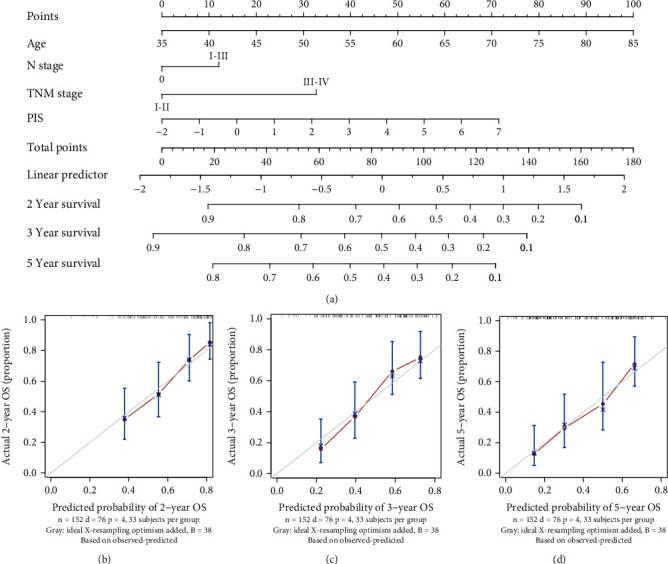
Nomogram predicts survival probability in ESCC patients. (a) Nomogram based on clinicopathological features (age, N stage, and TNM stage) and PIS predicts 2-, 3-, and 5-year survivals in ESCC patients. Nomogram evaluated by calibration curves of (b) 2 years, (c) 3 years, and (d) 5 years. The grey lines represent an ideal evaluation, whereas the blue lines represent the performance of the nomogram. PIS: prognostic immune score; OS: overall survival.

**Figure 5:**
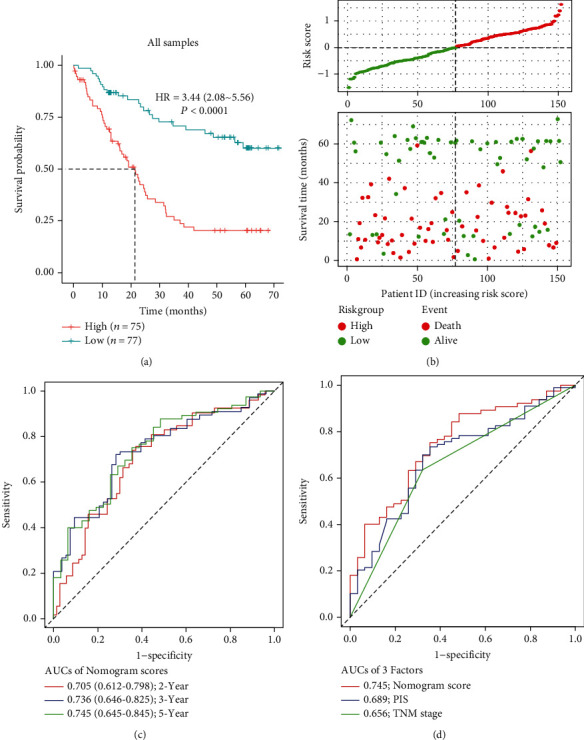
Prognostic performance of nomogram risk score. (a) Kaplan-Meier survival curve of patients with different nomogram risk score. (b) The nomogram risk score distribution. Green and red dots represent risk scores for low- and high-risk patients, respectively. The relationship between survival status and risk score. The abscissa represents the number of samples, and ordinate is the survival time. Red and green dots represent dead patients and alive patients. (c) The time-dependent AUCs of 2-, 3-, and 5-year nomogram risk score for all samples. (d) The time-dependent AUCs of TNM stage, PIS, and nomogram risk score on 5 years for all samples. Note: PIS: prognostic immune score.

The independent prognostic factors for overall survival of ESCC among clinicopathological features (gender, tobacco use, alcohol use, T stage, N stage, and TNM stage) as well as PIS were determined by univariate Cox regression analyses, and N stage and TNM stage were identified to be the independent factors associated with prognosis of ESCC (Supplemental Table 2). Seeking to improve the accuracy of prognostic classification, a prognostic nomogram model was constructed to incorporate independent clinicopathological features with prognostic relevance and PIS in the prognostic model (Figure 4(a)). The calibration curves for the nomogram of 2-, 3-, and 5-year survivals showed good agreement between prediction and the actual observation in all samples (Figures 4(b)–4(d)). The mean standard errors of 2-year, 3-year, and 5-year survivals were 0.146, 0.220, and 0.276, respectively. The Kaplan-Meier survival curves demonstrated that ESCC patients in the high-risk group had significantly worse overall survival than those in the low-risk group (HR = 4.17, 95% CI: 2.22 to 7.69, *P* < 0.0001, Figure 5(a)).  Figure 5(b) shows that the distribution of nomogram scores involved survival time and survival status, indicating good performance of the nomogram model. A time-dependent ROC analysis revealed the AUC for TNM stage, PIS, and nomogram model at 5-year were 0.656 (95% CI: 0.556 to 0.755), 0.689 (95% CI: 0.579 to 0.798), and 0.745 (95% CI: 0.644 to 0.845), respectively (Figures 5(c) and 5(d), Table 4). The 5-year AUCs of the nomogram model outperformed TNM stage or PIS as a prognostic model alone (*P* = 0.006 and *P* = 0.693, respectively), indicating that this nomogram model is a more reliable prognostic index.

## 4. Discussion

Esophageal cancer, including ESCC that is more prevalent in China, is clinically challenging and requires multidisciplinary care that comprises surgery, chemotherapy, radiotherapy, and immunotherapy [4]. Despite these efforts, recurrence and metastasis still ensue and render ESCC patient dismal clinical outcomes [4, 11]. Therefore, identification of a novel biomarker signature for diagnosis and prognosis as well as for therapeutic interventions holds promise for tailored care of ESCC. Based on the deconvolution of bulk transcriptome data of ESCC, 9 overlapping immune features identified by LASSO and Boruta algorithms were used for DIS calculation followed by effective discrimination of ESCC from normal esophageal mucosa tissue. Two immune cell types, namely, B naïve cells and plasma cells, identified by univariate and multivariate Cox proportional hazard regression analyses as independent prognostic factors, were used to construct a prognostic model that classify ESCC patients into high- and low-risk patients with significant differences in clinical outcomes. Furthermore, a nomogram model integrating age, N stage, TNM stage, and PIS shows robustness in predicative accuracy of prognosis for ESCC.

The initiation and development of malignancy are closely linked to inflammation, which fosters proliferation, survival, and migration during neoplastic progression [27, 28]. For example, elevated plasma levels of C-reactive protein are associated with reduced disease-free survival of breast cancer patients [29]. Furthermore, current smoking or prior heavy smoking that links to chronic lung inflammation is significantly associated with an increased risk of recurrence and mortality in breast cancer patients [30, 31]. In mice, lung inflammation induced by either tobacco smoke exposure or nasal instillation of lipopolysaccharide awakens dormant cancer cells [32]. Notably, chronic inflammation is an integral proportion of tumor microenvironment of ESCC evidenced by local infiltration of multiple immune cells and elevated circulated C-reactive protein [33]. In tumor microenvironment (TME), there exists a variety of immune and stromal cells that restrain or accelerate tumor growth. Mounting evidence indicates that infiltrating immune cell populations are associated with tumor growth, cancer progression, and clinical outcome in multiple cancers [34, 35]. Among the tumor-infiltrating immune cells, immune suppressor cells (T regulatory cells and M2 macrophage) are generally associated with poor prognosis, whereas cytotoxic T cells (CD8+ T cells, NK cells, and *γδ* T cells) are correlated with improved survival [11, 13–16, 28, 34, 35].

In recent years, various computational algorithms have been developed to estimate the immune components within TME using bulk transcriptome data [12, 17, 36–39]. The present study employed CIBERSORT to estimate the fractions of 22 immune cell subsets using transcriptome data from public domain. Among the ESCC and normal esophageal tissue samples with CIBERSORT *P* < 0.05, the proportions of 13 individual immune cell fractions in tumor tissues were significantly different from those in normal tissues. In TME of ESCC, the immunostimulating cells including *γδ* T cells, mast cells, and B cells were underrepresented, whereas the immunosuppressive cells, including M0 and M1 macrophages and neutrophil, were more abundant in ESCC compared with normal esophageal tissues. By leveraging these different immune features with XGBoost, we built a DIS that distinguished ESCC from normal squamous mucosa with reliable accuracy. Our results demonstrate that tumor immune infiltrates play oncogenic roles in pathogenesis and progression of ESCC and serve as novel potential biomarkers for detection and diagnosis of ESCC.

Although CIBERSORT *P* values represent the total infiltrated immune cells in TME, no prognostic effect was observed in the highly infiltrated subset (CIBERSORT *P* < 0.01) compared with the subset lacking immune infiltration (CIBERSORT *P* > 0.05) in the context of ESCC (Supplemental Figure 4(b)). Nevertheless, a total of 8 immune cells including naive B cells, CD8 T cells, regulatory T cells, gamma delta T cells, activated NK cells, M0 macrophages, resting dendritic cells, and resting mast cells were significantly correlated with clinical outcomes of ESCC patients by the univariate Cox regression analysis. Notably, ESCC patients with higher fractions of M0 macrophage showed poorer overall survival (Supplemental Figure 4(c)). Tumor-associated macrophages (TAM), the major component in TME, are functionally classified in two different subtypes, i.e., M1 and M2 macrophages, which show distinct effector molecules on plasma membrane. Generally, M1 and M2 macrophages assume tumoricidal and protumor functions, respectively, in the evolution of malignancy [40, 41]. Higher proportions of M2 macrophages contribute to an immunosuppressive microenvironment and have been associated with therapeutic resistance and poor prognosis in multiple cancers, including both ESCC and EAC [11, 42, 43]. Targeting macrophages, in particular M2 macrophages, improved antitumor immunity through reprograming of immune cells [44]. In line with this, we also found negative correlation between ESCC prognosis and M0 and M2 macrophages, indicating the skewed differentiation of M0 towards M1 polarization. Additionally, higher proportions of resting memory CD4 and *γδ* T cells, in addition to M0 and M2 macrophages, were also found to be negative prognostic markers of clinical outcome. In contrast, greater infiltration of plasma cells, CD8 T cells, activated NK cells, and resting mast cells was correlated with improved prognosis. In immune-oncology, the cytotoxicity exerted by T and NK cells has been well recognized and received the greatest attention. On the other hand, the role of B lymphocytes has begun to be appreciated in the context of host-tumor interaction over the last decade. Through antibody-dependent cell cytotoxicity and complement cascade activation, B and plasma cells can kill cancer cells and are correlated with improved cancer outcome. In contrast, tumor-promoting roles have also been found in multiple cancers [42, 45, 46]. Based on the prognostic relevance of immune features generated by CIBERSORT, multivariable Cox regression approach was used to select the key prognostic features for PIS building. The ESCC patients with low PIS have favorable outcomes compared with those with high PIS. To further improve the predicative accuracy for prognosis, we also established a nomogram model, which integrate age, N stage, TNM stage, and PIS, with improved performance compared with PIS and TNM stage as a prognostic model alone.

On aggregate, our data argue that the infiltrated immune populations in TME are heterogeneous in terms of phenotype and function, which play divergent roles in the development and progression of ESCC. The dichotomy in immune functions was supported by the evidence of negative or positive correlations with cytolytic activity, which was closely correlated with expression of GZMA, GZMK, and PRF1 [47]. Thus, the functional state of immune cells in TME per se, rather than the abundance, is the determinant of immune response against cancer.

Although both ESCC and EAC (some cases if not all) arise from esophageal squamous epithelium, they have remarkable distinctions in terms of histology, geographic patterns, time trends, etiological factors, and molecular features [7, 48]. Furthermore, tumor immune infiltrates in ESCC are completely different from those in EAC [49]. Strikingly, genetic studies revealed that multiple mutation-associated driver genes are shared among ESCC, lung squamous cell carcinoma, and head and neck squamous cell carcinoma [7]. Nevertheless, several previous studies that explored the roles of tumor immune infiltrates did not separate ESCC from EAC. Our PIS for ESCC failed to discriminate EAC with diverse clinical outcomes, further supporting that ESCC and EAC are histologically and molecularly distinct diseases and arguing against the combination of ESCC and EAC as esophageal cancer for mechanistic investigation and clinical trials. This is the main strength of this study.

Our study also has limitations. As we all know, ESCC is prevalent in China contrasting with EAC more frequent in western countries. Nevertheless, genetic aberrations are remarkably distinct between ESCC cases from USA and southern China [50–52]. Furthermore, American ESCC that occurs more common in blacks compared with whites in the United States [53] shares only 30% differential gene expression with Chinese ESCC, indicating that demographic factors such as genetic ancestry could account for variation of genetic phenotype. Therefore, our PIS derived from Chinese ESCC is not likely feasible for ESCC from other sources, especially western countries. This is the main limitation of this study. Second, in this work, all data were from public databases and the clinical utility of our DIS and PIS was not verified in independent clinical ESCC samples. Third, some factors, including living environment, drinking habits, family history, and microbial infection, were incomplete for ESCC patients in this study, which might underestimate the value of our diagnostic and prognostic models.

## 5. Conclusion

In summary, the present study demonstrates the diagnostic and prognostic potential of our DIS and PIS based on the differential distribution of infiltrated immune cells enumerated by deconvolution of transcriptome. A nomogram model integrating clinicopathological characteristics and immune signature shows improved accuracy for prognostic classification over TNM stage or PIS as a prognostic alone, which warrants prospective studies to validate.

## Figures and Tables

**Table 1:** Demographic and clinicopathological characteristics of eligible patients from GSE53625 and TCGA-ESCC.

Characteristic		GSE53625 (*n* = 101)	TCGA-ESCC (*n* = 51)
Gender (%)	Female	23 (22.8)	4 (7.8)
	Male	78 (77.2)	47 (92.2)
Age (mean (SD))		59.19 (8.1)	57.67 (9.5)
Tobacco use (%)	No	38 (37.6)	20 (39.2)
	Yes	63 (62.4)	30 (58.8)
	NA	0 (0)	1 (2.0)
Alcohol use (%)	No	44 (43.6)	16 (31.4)
	Yes	57 (56.4)	35 (68.6)
T stage (%)	I-II	80 (79.2)	19 (37.3)
	III-IV	21 (20.8)	32 (62.7)
N stage (%)	No	49 (48.5)	28 (54.9)
	Yes	52 (51.5)	23 (45.1)
TNM stage (%)	I-II	49 (48.5)	31 (60.8)
	III-IV	52 (51.5)	20 (39.2)

**Table 2:** Different proportions of immune cells and scores determined by CIBERSORT and ESTIMATE algorithms, respectively, in normal and tumor tissues of ESCC.

Elements	Tumor mean	Normal mean	Fold change (T/N)	*P* value
*Naive B cells*	**0.0092**	**0.0298**	**0.3087**	**0.0005**
*Memory B cells*	**0.0165**	**0.0324**	**0.5093**	**0.0021**
*Plasma cells*	**0.0955**	**0.1245**	**0.7671**	**0.0072**
CD8 T cells	0.1339	0.1459	0.9177	0.1571
Naive CD4 T cells	0.0011	2.6*E* − 05	NA	0.1250
Resting memory CD4 T cells	0.0846	0.0994	0.8511	0.0584
*Activated memory CD4 T cells*	**0.0658**	**0.0141**	**4.6667**	1.3**E** − 08
Follicular helper T cells	0.0165	0.0141	1.1702	0.7114
*Regulatory T cells (Tregs)*	**0.0295**	**0.0435**	**0.6782**	**0.0011**
*Gamma delta T cells*	**0.0101**	**0.0312**	**0.3237**	8.6**E** − 06
*Resting NK cells*	0.0014	0	NA	0.0365
Activated NK cells	0.0630	0.0703	0.8962	0.0629
*Monocytes*	**0.0525**	**0.0942**	**0.5573**	8.9**E** − 07
*M0 macrophages*	**0.0776**	**0.0124**	**6.2581**	3.4**E** − 16
*M1 macrophages*	**0.1169**	**0.0396**	**2.9520**	2.0**E** − 16
M2 macrophages	0.0897	0.0798	1.1241	0.2369
Resting dendritic cells	0.0378	0.0385	0.9818	0.5005
Activated dendritic cells	0.0070	0.0011	6.3636	0.0501
*Resting mast cells*	**0.0621**	**0.1015**	**0.6118**	6.5**E** − 07
Activated mast cells	0.0151	0.0092	1.6413	0.7812
*Eosinophils*	**0.0112**	**0.0181**	**0.6188**	**0.0051**
*Neutrophils*	**0.0030**	**0.0002**	**15.0010**	**0.0045**
*StromalScore*	**0.5942**	**0.3689**	**1.6107**	**0.0032**
*ImmuneScore*	**1.1312**	**1.3004**	**0.8699**	**0.0118**
ESTIMATEScore	1.7255	1.6693	1.0337	0.9394

Note: *P* values were calculated by the Wilcoxon-signed rank test.

**Table 3:** Performance metrics of diagnostic immune score for discrimination of tumor from normal tissue in different datasets.

Dataset	Sn	Sp	Acc	AUC
Training cohort (GSE53625)	1.000	0.981	0.993	0.999
Internal validation cohort (GSE23400)	0.717	0.875	0.792	0.813
External validation cohort (TCGA-ESCC and GTEx)	0.843	0.960	0.882	0.966

Note: Sn: sensitivity; Sp: specificity; Acc: overall accuracy.

**Table 4:** Time-dependent AUCs of overall survival with TNM stage, prognostic immune score (PIS), and nomogram score for all samples.

	AUCs (95% CI)		
	2 years	3 years	5 years
TNM stage	0.649 (0.564-0.734)	0.661 (0.575-0.747)	0.656 (0.557-0.756)

PIS	0.595 (0.492-0.695)	0.642 (0.541-0.745)	0.689 (0.579-0.798)

Nomogram score	0.705 (0.612-0.799)	0.736 (0.646-0.825)	0.745 (0.644-0.845)

## Data Availability

The datasets presented in this study can be found in online repositories (TCGA, GTEx, and GEO), and scripts are available on request.

## Abbreviations

EC: Esophageal cancer
ESCC: Esophageal squamous cell carcinoma
EAC: Esophageal adenocarcinoma
GEO: Gene Expression Omnibus
TCGA: The Cancer Genome Atlas
GTEx: Genotype-Tissue Expression
CIBERSORT: Cell type identification by estimating relative subsets of RNA transcripts
TPM: Transcripts per kilobase of exon model per million mapped reads
TIIC: Tumor immune infiltration cell
DIS: Diagnostic immune score
PIS: Prognostic immune score
LASSO: Least absolute shrinkage and selection operator
XGBoost: eXtreme gradient boosting
ROC: Receiver operating characteristic
AUC: Area under the ROC curve
CI: Confidence interval.

## Supplementary Materials

Supplementary Materials Supplementary Table 1: the univariate Cox regression analysis of 8 immune cells and StromalScore in the training cohort. Supplementary Table 2: the univariate Cox regression analysis of demographic and pathological features in the training cohort. Supplementary Figure 1: distribution of 22 infiltrated immune cells in 78 normal tissues and 152 ESCC tissues. Each bar represents the relative proportions of infiltrated immune cells of each subject. Supplementary Figure 2: box plot of immune cells between normal and tumor tissues in different groups, separated by gender, tobacco use, and alcohol use. ns: *P* ≥ 0.05; ∗: *P* < 0.05; ∗∗: *P* < 0.01; ∗∗∗: *P* < 0.001. Supplementary Figure 3: box plot of immune cells between normal and tumor tissues in different groups, separated by T stage, N stage, and TNM stage. ns: *P* ≥ 0.05; ∗: *P* < 0.05; ∗∗: *P* < 0.01; ∗∗∗: *P* < 0.001. Supplementary Figure 4: Kaplan-Meier curves for overall survival. (a) EAC samples in TCGA-EAC grouped by PIS. (b) ESCC samples in GSE53625 grouped by CIBERSORT *P* value. (c) ESCC samples in the training cohort grouped by immune cell percentage of M0 macrophages. Note: *P* values were calculated by log-rank test. HR: hazard ratio; PIS: prognostic immune score.
