# Supplementary material for: Immune Infiltration Represents Potential Diagnostic and Prognostic Biomarkers for Esophageal Squamous Cell Carcinoma
Source: Biomed Res Int. 2022 Jun 26;2022:9009269. doi: 10.1155/2022/9009269 (PMC9251101; doi:10.1155/2022/9009269)
Supplement: Supplementary Materials — Supplementary Table 1: the univariate Cox regression analysis of 8 immune cells and StromalScore in the training cohort. Supplementary Table 2: the univariate Cox regression analysis of demographic and pathological features in the training cohort. Supplementary Figure 1: distribution of 22 infiltrated immune cells in 78 normal tissues and 152 ESCC tissues. Each bar represents the relative proportions of infiltrated immune cells of each subject. Supplementary Figure 2: box plot of immune cells between normal and tumor tissues in different groups, separated by gender, tobacco use, and alcohol use. ns: P ≥ 0.05; ∗: P < 0.05; ∗∗: P < 0.01; ∗∗∗: P < 0.001. Supplementary Figure 3: box plot of immune cells between normal and tumor tissues in different groups, separated by T stage, N stage, and TNM stage. ns: P ≥ 0.05; ∗: P < 0.05; ∗∗: P < 0.01; ∗∗∗: P < 0.001. Supplementary Figure 4: Kaplan-Meier curves for overall survival. (a) EAC samples in TCGA-EAC grouped by PIS. (b) ESCC samples in GSE53625 grouped by CIBERSORT P value. (c) ESCC samples in the training cohort grouped by immune cell percentage of M0 macrophages. Note: P values were calculated by log-rank test. HR: hazard ratio; PIS: prognostic immune score. [file 9009269.f1.docx]

Immune Infiltration Represents Potential Diagnostic and Prognostic Biomarkers for Esophageal Squamous Cell Carcinoma

**Supplementary Materials**

**Supplementary Table 1** The univariate Cox regression analysis of 8 immune cells and StromalScore in training cohort.

| Cell type | HR (95%CI) | *P* value |
| --- | --- | --- |
| B cells naive | 0.50 (0.26 - 0.96) | 0.032 |
| B cells memory | 1.20 (0.59 - 2.44) | 0.607 |
| Plasma cells | 3.40 (0.82 - 14.06) | 0.073 |
| T cells CD4 memory activated | 1.53 (0.84 - 2.79) | 0.163 |
| Monocytes | 0.63 (0.33 - 1.19) | 0.149 |
| Macrophages M0 | 0.46 (0.24 - 0.91) | 0.021 |
| Macrophages M1 | 1.73 (0.87 - 3.44) | 0.114 |
| Mast cells resting | 2.40 (1.15 - 5.03) | 0.016 |
| StromalScore | 0.31 (0.14 - 0.69) | 0.002 |

**Supplementary Table 2** The univariate Cox regression analysis of demographic and pathological features in training cohort.

| Feature | HR (95%CI) | P value |
| --- | --- | --- |
| Gender | 0.95 (0.46-1.96) | 0.8919 |
| Tobacco use | 1.07 (0.55-2.07) | 0.8375 |
| Alcohol use | 1.68 (0.87-3.26) | 0.1184 |
| T stage | 1.94 (0.81-4.65) | 0.1295 |
| **N stage** | **3.01 (1.51-5.99)** | **0.0010** |
| **TNM stage** | **3.67 (1.81-7.46)** | **0.0001** |

**Supplementary Figure Legends**

**
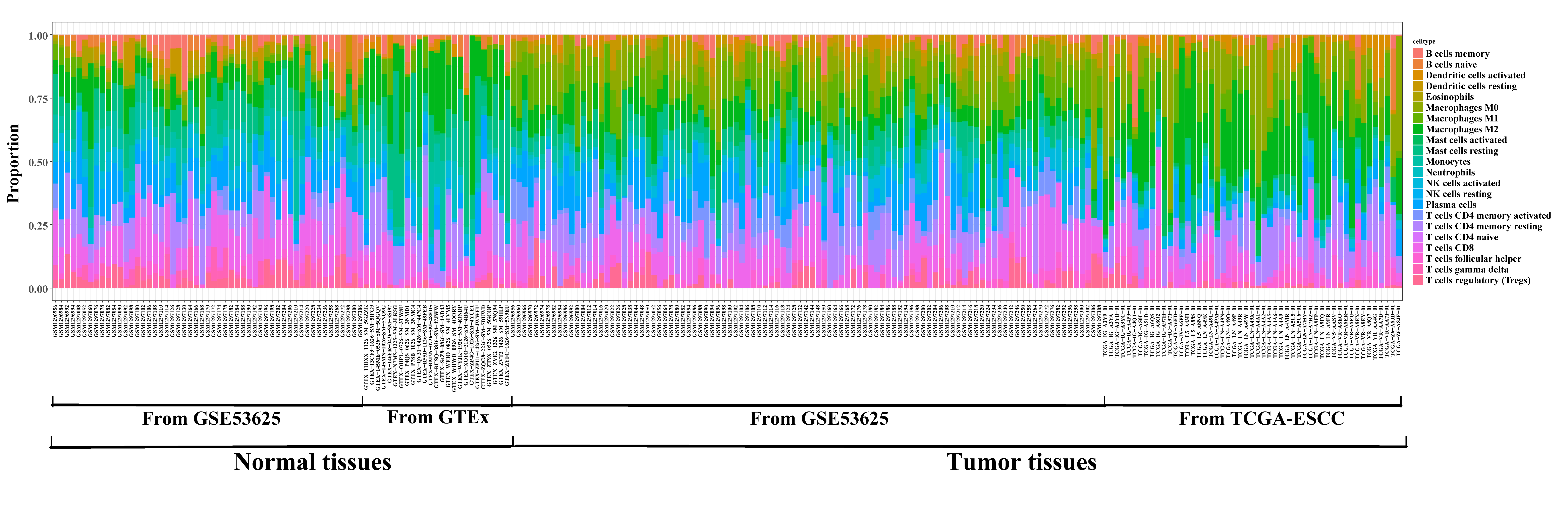
**

**Supplementary Figure 1** Distribution of 22 infiltrated immune cells in 78 normal tissues and 152 ESCC tissues. Each bar represents the relative proportions of infiltrated immune cells of each subject.


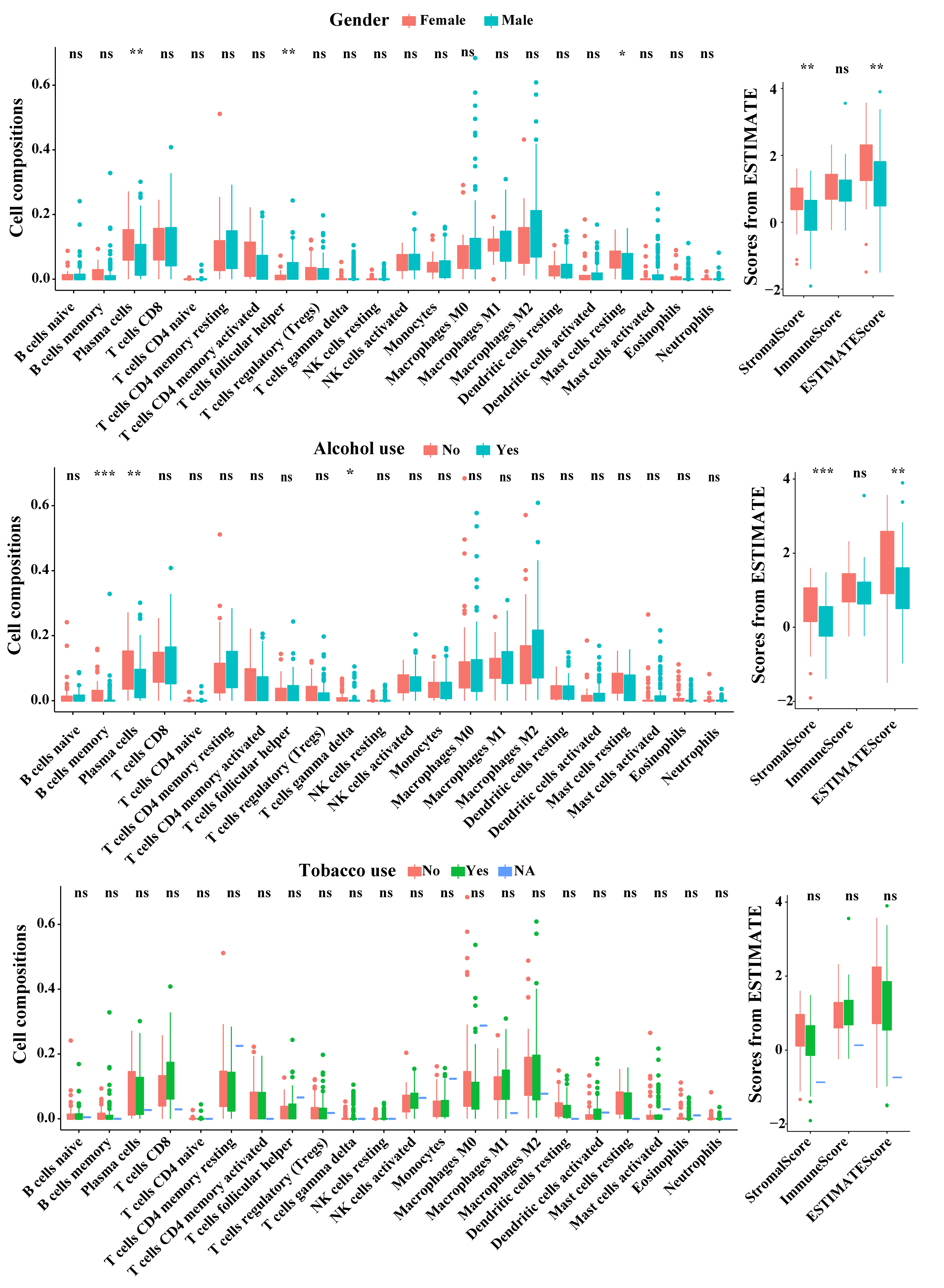


**Supplementary Figure 2** Box plot of immune cells between normal and tumor tissues in different groups, separated by gender, tobacco use, and alcohol use. ns: *P>*=0.05; *: *P*<0.05; **: *P*<0.01; ***: *P*<0.001.


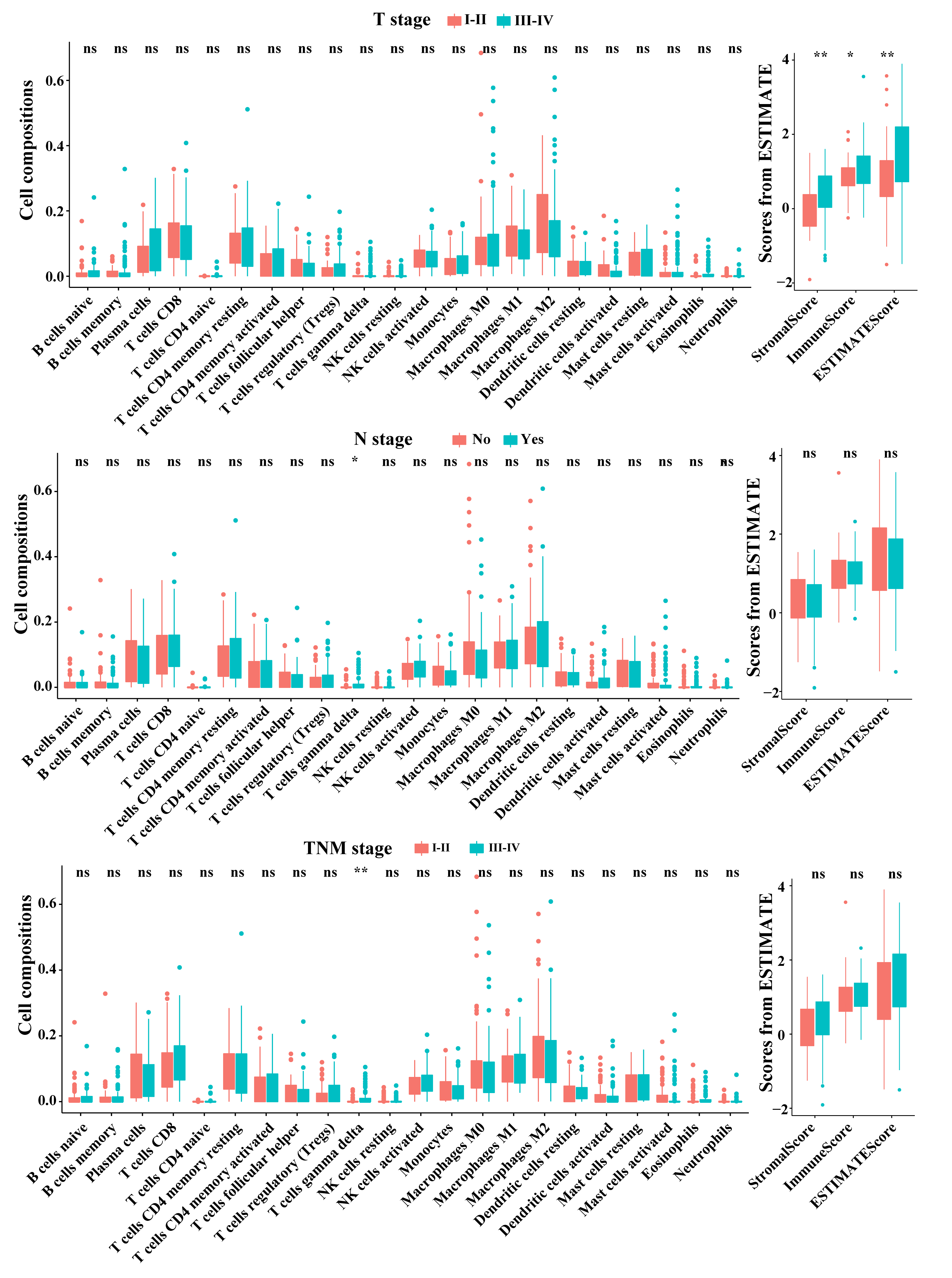


**Supplementary Figure 3** Box plot of immune cells between normal and tumor tissues in different groups, separated by T stage, N stage, and TNM stage. ns: *P>*=0.05; *: *P*<0.05; **: *P*<0.01; ***: *P*<0.001.


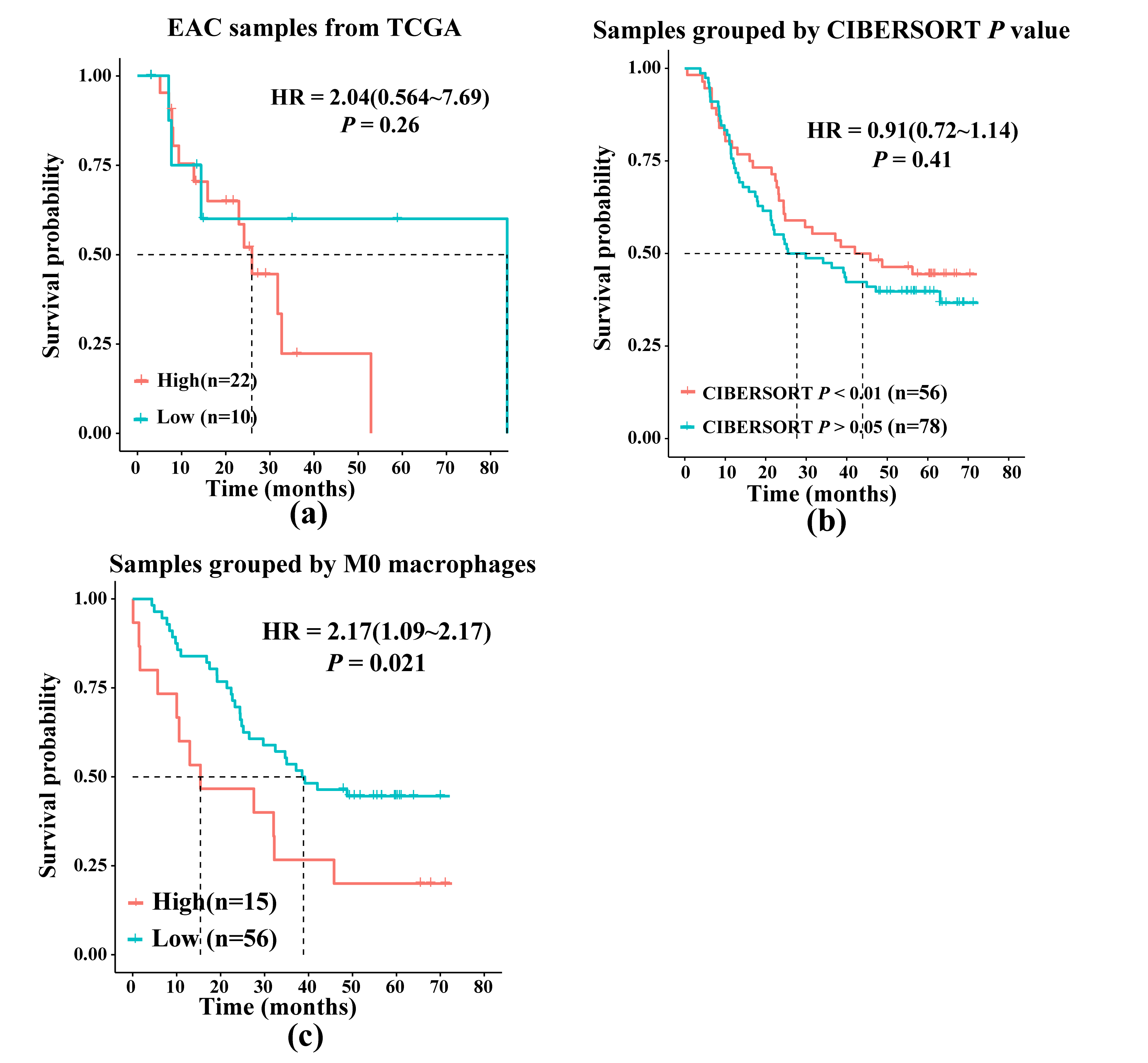


**Supplementary Figure 4** Kaplan-Meier curves for overall survival. (a) EAC samples in the TCGA-EAC grouped by PIS. (b) ESCC samples in GSE53625 grouped by CIBERSORT *P* value. (c) ESCC samples in the training cohort grouped by immune cell percentage of M0 macrophages. *P* values were calculated by log-rank test. HR, hazard ratio. Note: PIS, prognostic immune score.
